# Guillain–Barré Syndrome Secondary to West Nile Virus in New York City

**DOI:** 10.1155/2020/6501658

**Published:** 2020-07-26

**Authors:** Rafail Beshai, Daniel Bibawy, Joseph Bibawy

**Affiliations:** ^1^Liberty College of Osteopathic Medicine, Lynchburg, VA, USA; ^2^New York Institute of Technology College of Osteopathic Medicine, Old Westbury, NY, USA; ^3^Department of Medicine, Richmond University Medical Center, Staten Island, NY, USA

## Abstract

West Nile virus (WNV) is an arthropod-borne flavivirus belonging taxonomically to the Japanese encephalitis subgroup. Usually, it is transmitted by *Culex pipiens* mosquitoes. Consequently, an increase in WNV-positive mosquitoes presents a rise of the number of patients, as it has been seen in NYC. We present a 65-year-old patient with WNV infection who presented with Guillain–Barré syndrome (GBS). She had a rapidly progressing ascending paralysis, a common feature in GBS patients but an uncommon presentation in WNV. Realizing WNV as an emerging pathogen along with its uncommon presentation of GBS can be potentially lifesaving if caught at an early stage.

## 1. Introduction

Guillain–Barré syndrome (GBS) was first described by Guillain, Barré, and Strohribed in 1916 [1]. It is a rare disease with an incidence rate of 1 in 100000 [2]. It manifests as a bilateral weakness of the extremities that usually starts in the lower limb and ascends up toward arms and facial muscles later in the course of the disease [3]. The weakness may vary greatly from difficulty walking to complete paralysis. Severe respiratory muscle weakness and decreased deep tendon reflexes are also common presentations [3, 4]. Risk factors include male sex, older age, and infection with *Campylobacter jejuni* or Epstein–Barr virus [5]. Two-thirds of patients have a previous respiratory or digestive infection as can be seen in our case [6]. The condition is difficult to diagnose because it may involve specialist consultations (e.g., neurology) or procedures (e.g., lumbar puncture and nerve conduction studies). Cerebrospinal fluid usually shows elevated proteins with normal white blood cell count [7]. There are different proposed mechanisms for the pathophysiologic process of GBS. However, the most accepted mechanism is that of molecular mimicry which means that there is cross reaction with epitopes on peripheral nerve after an initial immune reaction against an infection [8]. The activated T cells will produce different cytokines that will recruit macrophages which will damage the myelin sheath. This damage will lead to decrease in conduction velocities affirmed by nerve conduction studies [8, 9]. There is another mechanism where the T cells attack the axons itself instead of the myelin sheath but it is not common in the United States. Management and treatment of patients with GBS involves both supportive medical care and immunotherapy. The supportive medical care is critical because 30% of patients will develop respiratory failure requiring ventilation [10]. In addition to that, close monitoring of cardiac and hemodynamic functioning is important [10]. The immunotherapy treatments include IVig that inhibits Fc-mediated activation of immune cells and plasma exchange which removes neurotoxic antibodies from the body [8, 11]. Both will eventually lead to decrease in the destruction of myelin sheath. The in-hospital mortality rate of GBS is only 2.6%, but still the treatment has better outcomes if the disease is discovered early [4]. We report an unusual presentation of Guillain–Barré syndrome that follows an infection with WNV. According to CDC, only 13% of WNV infection leads to GBS [12]. As a result, GBS should be in the differential diagnosis after a WNV infection.

## 2. Case Report

A 65-year-old Hispanic woman presented to the emergency department with a 1-week history of intermittent fever. She was seen by an emergency room doctor at that time and was sent home after being diagnosed with pneumonia after a chest radiograph was performed showing a questionable left lung base infiltrate and discharged with levofloxacin and ibuprofen. The patient returned to the emergency department 3 days later because of worsening symptoms described as weakness and inability to walk coupled with fever of 101 degrees Fahrenheit. As per the family, the patient was unusually confused, had difficulty with comprehension, and articulating her needs. In addition to these symptoms, the patient denied any other constitutional symptoms, sick contact, recent travel, or interaction with pets or animals. A thorough physical examination revealed crackles in the right middle and lower lungs; therefore, another chest radiograph was performed showing increased patchy density at the left lung base as compared with prior exam. She was started on 1 gram of ceftriaxone daily and 500 mg of azithromycin daily for treatment of the pneumonia at this time. A series of neurological examinations initially revealed a mildly confused elderly woman who was, nonetheless, oriented to person, time, and place. The patient stated she felt weak but was able to move her legs. She could move her arms but could not raise them more than approximately 10 inches. Consequently, the neurologist ordered the first CT scan which had unremarkable findings. On day 2 of admission, the patient was awake, alert, but oriented only to person. She denied headache but had a rigid neck with her speech becoming increasingly incomprehensible although she was able to follow few simple commands. Neurological exam was significant for asymmetric facial droop and left arm dropping fast than right and drop arm test. Stroke notification was called, and another noncontrast CT of the head was performed which was unremarkable.

Later on the second day of admission, she became unresponsive and required intubation and was transferred to the Medical Intensive Care Unit (MICU). She only responded to painful stimuli by moving all extremities. For the next two days, the neurological examination showed a rapidly progressing ascending paralysis with decreased deep tendon reflexes of the upper and lower extremities. During her stay in the MICU, subsequent chest radiographs and respiratory, blood, and urine cultures were acquired, and a lumbar puncture was performed. She was started on treatment for suspected bacterial and viral meningitis with azithromycin, ampicillin, ceftriaxone, and vancomycin while awaiting test results. Acyclovir was also administered due to suspicion of a herpes simplex encephalitis. Dexamethasone and IViG were administered for suspicion of acute disseminated encephalomyelitis. Respiratory cultures were recovered from an endotracheal aspirate revealing light yeast formation. CSF obtained from lumbar puncture was positive for elevated proteins and normal white blood cell count. Considering the unique CSF findings, Guillain–Barré Syndrome (GBS) was considered, and its etiology was invested. Nerve conduction studies and an electromyography (EMG) were performed revealing acute sensorimotor axonal and demyelinating peripheral neuropathy consistent with GBS. Serology tests were subsequently ordered and West Nile virus IgM was reported positive. CSF serology was tested for Lyme and herpes simplex virus which were negative. Urine was also tested for *Legionella* sp. which resulted negative. Findings were reported to the Department of Health (DOH), and the case was discussed with hospital medical team and family members.

Given the patient's presentations and the serological tests, it was determined that GBS secondary to WNV infection was most likely the diagnosis. Treatment for meningitis was discontinued, and intravenous immunoglobulin was continued for 10 days and plasmapheresis would be considered thereafter. After 4 days of 30 mg IViG, she showed substantial improvement moving her toes and closing her fist. At 10 days, she was able to relay requests with improved concentration and without facial droop, and also muscle strength improved on a daily basis. After completion of 10 days of IViG, no further plasmapheresis was initiated considering her continued improvement and supportive care was recommended. The hospital course was complicated by hyponatremia and serum hypo-osmolality considered secondary to Syndrome of Inappropriate Antidiuretic Hormone Secretion (SIADH). She was treated with fluid restriction and followed closely by the nephrology specialist adjusting her fluid intake daily. She remained in the hospital until hyponatremia was corrected and maintained. The total hospital course was 20 days, and she was thereafter transferred to a rehab facility where she continued to improve. As of the time this paper is being written, the patient has regained full functionality of her upper extremities and is no longer requiring ventilator support. Nonetheless, lower extremity weakness has remained unchanged, unfortunately.

## 3. Discussion

West Nile virus is a member of the flaviviridae family that is primarily transmitted to humans by mosquitoes. Although at first not endemic to North America, outbreaks in New York and other areas of the United States from 1999–2012 have led to its evolution in North America and is now considered to be endemic to North America [13]. From 2014 to 2018, it was found that Staten Island had a high quantity of standing water, which attracts mosquitoes [14]. Furthermore, mosquitoes carrying West Nile virus were found in that same time period when the patient first presented to the emergency department [14]. Most patients infected with WNV are asymptomatic, but 30 to 40% will have symptoms that range from fatigue, memory impairment, weakness, and headache and balance problems to meningitis, encephalitis, flaccid paralysis, and photophobia [15]. Age, malignancy, and genetics are factors that increase the neuroinvasiveness of WNV [15]. Diagnosis of WNV is difficult because it needs serological tests and PCR. As stated previously, the CDC reports that 13% of West Nile virus infection will present with GBS [12]. In about 1/3 of patients, WNV can lead to mild hyponatremia, compatible with the syndrome of inappropriate antidiuretic hormone secretion [16]. However, treating this patient with IVig for GBS in addition to WNV infection put her in an increased risk factors for SIADH [17]. This is likely the case in this patient presentation, as she responded well to fluid restriction. This case report highlights the importance of realizing West Nile virus as a pathogen during summer time especially in places where high quantity of standing water is present. In particular, it serves as an alert to clinicians in regions with high number of mosquitos of the potential risk 9 for GBS and SIADH and the need for timely detection, diagnosis, and initiation of treatment and supportive care to prevent mortality and long-term sequelae.

Although further studies of early diagnosis for West Nile virus infection are warranted, we propose here that putting WNV in your differential diagnosis could be lifesaving. GBS is known to be commonly preceded by a number of agents, such as varicella zoster virus, herpes simplex virus, hepatitis A, B, C, and E viruses, human immunodeficiency virus (HIV), and *Mycoplasma pneumoniae* [5]. Some of these agents, particularly HIV and *M. pneumoniae*, are also known to cause pneumonia [18]. In our patient, the respiratory cultures collected from endotracheal aspirates found yeast early in her hospital stay. On day 12 of the patient's hospital stay, endotracheal respiratory cultures revealed *Staphylococcus* coagulase negative indicating *Staphylococcus* (S.) *aureus* as new source of her pneumonia. Although *S. aureus* was present in cultures, it is unlikely to be the original agent in the patient's pulmonary compromise and is considered a hospital-acquired pneumonia. While it is possible that the antecedent to this patient's GBS was *S. aureus*, it seems more likely that her WNV infection is what led to the development of GBS, as there is a lack of research linking *S. aureus* to the development of GBS. Furthermore, weakness from the developing neurological deficits is more likely to have led to the development of poor respiratory effort resulting in pneumonia, as opposed to the pneumonia being the etiology of the neurological disease. Further research connecting WNV to GBS is necessary to make a definitive conclusion on which of this patient's infections led to her development of GBS. However, we believe that in this case, the patient's diagnosis of West Nile virus was the agent that led to her later complication of Guillain–Barré Syndrome.

## Data Availability

The data supporting the findings of this study will be made available from the corresponding author upon request (josephbibawy@gmail.com).
